# A Review on Microdevices for Isolating Circulating Tumor Cells

**DOI:** 10.3390/mi11050531

**Published:** 2020-05-22

**Authors:** Kin Fong Lei

**Affiliations:** 1 Graduate Institute of Biomedical Engineering, Chang Gung University, Taoyuan 333, Taiwan; kflei@mail.cgu.edu.tw; Tel.: +886-3-2118800 (ext. 5345); 2 Department of Radiation Oncology, Chang Gung Memorial Hospital, Linkou 333, Taiwan

**Keywords:** cancer metastasis, circulating tumor cells, CTC isolation, microdevices

## Abstract

Cancer metastasis is the primary cause of high mortality of cancer patients. Enumeration of circulating tumor cells (CTCs) in the bloodstream is a very important indicator to estimate the therapeutic outcome in various metastatic cancers. The aim of this article is to review recent developments on the CTC isolation technologies in microdevices. Based on the categories of biochemical and biophysical isolation approaches, a literature review and in-depth discussion will be included to provide an overview of this challenging topic. The current excellent developments suggest promising CTC isolation methods in order to establish a precise indicator of the therapeutic outcome of cancer patients.

## 1. Introduction

According to clinical reports, cancer metastasis is the primary cause of high mortality of cancer patients [1,2]. Cancer metastasis is a multistep process of tumor cells escaping from the primary tumor site, entering into the bloodstream, arresting at the secondary site, extravasating into the tissue, and forming secondary tumor colonies [3,4]. During this process, the tumor cells that circulate in the bloodstream are called circulating tumor cells (CTCs). Because tissue biopsies are difficult to obtain from patients for evaluating the response of cancer therapy, CTCs are recognized as liquid biopsies to collect tumor cells from blood. The CTCs were first discovered from a cancer patient by Ashworth in 1869 [5]. Although it is still unclear exactly when and how the metastatic process begins and which factors drive the process, it was confirmed that CTCs are associated with poor progression-free and overall survival [6,7,8]. In most cases of cancer patients, metastasis, not the primary tumor, causes the death of the patient. Additionally, when a patient is diagnosed with cancer before spreading outside the primary site, the survival chance can be highly improved. However, because of the detection limit of physical examination and traditional imaging methods such as magnetic resonance imaging (MRI), positron emission tomography (PET), computerized tomography (CT), X-ray and ultrasound, it is difficult to detect small metastasis. For example, the detection limit of breast cancer tumors using ultrasound and mammography is 6 mm or larger [9]. Small lesions or micro-metastases are difficult to spot. On the other hand, blood withdrawal is a minimally invasive clinical process and is acceptable to cancer patients. The isolation of CTCs from blood samples can obtain tumor cells for evaluating the cancer progression or the response to cancer therapy. Nowadays, it is generally believed that CTCs play an important role in cancer metastasis [10,11,12,13,14]. Thus, the enumeration of CTCs was established to be an independent prognostic factor for survival. Clinical evaluations in breast, colon, and prostate cancer revealed that the presence of CTCs in 7.5 mL blood strongly correlates with progression-free and overall survival [6,7,8]. Thus, CTCs came to serve as a biomarker for evaluating cancer progression and the response to cancer therapy [10,11,12,13,14,15,16,17].

There is no doubt that the research of CTC isolation has become an attractive and important topic because it has significant clinical implications. However, CTC isolation from blood samples is technically challenging because the CTCs are extremely rare and hide in other circulating cells, e.g., erythrocytes and leukocytes. It was reported that CTCs in the peripheral blood of patients with metastatic disease range from 0 to 10,000 CTCs per mL of whole blood [18]. Moreover, they are obscured by billions of peripheral blood cells. Comparably, the isolation of erythrocytes is easy because of distinct physical, chemical, and biological properties. However, leukocytes and CTCs share many common properties. Development of effective CTC isolation technologies is still challenging but important. To evaluate the performance of the isolation technologies, it is important to note the following three design objectives: (1) high capture efficiency (isolate all of the CTCs in the blood sample); (2) high isolation purity (isolate only the CTCs in the blood sample); (3) high throughput (perform large volume of blood sample in a reasonably short time).

The aim of this review article is to summarize the recent developments on microdevices for isolating CTCs. The principles of CTC isolation can be generally divided into two approaches, namely biochemical and biophysical approaches. Microdevices are adopted with one or both approaches to accomplish CTC isolation. A summary of the CTC isolation microdevices is shown in Table 1. To compare the capture efficiency, isolation purity, and throughput of different isolation principles, a summary is listed in Table 2. A literature review and in-depth discussion of these microdevices are included in this review article. These developments provide a solid foundation to achieve a promising CTC isolation in order to establish a precise indicator of therapeutic outcome for cancer patients.

## 2. CTC Isolation Principles

Generally, CTC isolation principles are categorized into biochemical and biophysical approaches [31]. The former approach is based on the recognition of unique biomarkers of CTCs and the latter approach relies on the differentiation of the physical properties of CTCs and blood cells. In this section, a brief overview is provided for background information.

### 2.1. Biochemical Approach

Today, the understanding of cancer metastasis is still far from complete [3,4]. Although all CTCs go through the process of exiting from the primary tumor site and intravasting into the bloodstream, the expression of their surface targets may vary among malignant cells. Cancer biologists are working hard to find unique and well-defined universal surface targets to determine all malignant cell types. Currently, CTC isolation technologies mostly use epithelial marker, i.e., epithelial cell adhesion molecule (EpCAM), to isolate CTCs from blood sample. Although this approach may miss a critical subpopulation [32], a commercial CellSearch system uses EpCAM antibodies to capture CTCs. It is the only analytically valid and FDA-approved platform for prognostic use in breast, prostate, and colorectal cancers [33,34,35]. The CellSearch isolates CTCs in whole blood by using anti-EpCAM antibodies conjugated to magnetic beads. Magnetic beads are iron oxide particles encapsulated with polymers ranging from 35 nm up to 4.5 μm. One of the major advantages is they can be manipulated remotely by magnetism. The surface of these micro/nano-sized particles can be modified to immobilize antibodies; therefore, the target antigens suspending in liquid can be captured by functionalized magnetic beads in three-dimensional space. That highly improves the detection limit, especially for targets in extremely low concentrations. After magnetic capture, CTCs are identified by the staining of nuclei (DAPI), epithelial structural cytokeratins (CK8, CK18, and CK19), and anti-CD45 for differentiating from blood cells. In addition, in order to sample a larger blood volume, a medical wire functionalized with anti-EpCAM antibody was developed [36,37,38]. It is inserted into the cubital vein of a patient for 30 min. After the medical wire is removed, CTCs are identified and counted by staining of EpCAM, cytokeratins, and nuclei. The above biochemical approaches are based on detecting the surface targets of CTCs, such as EpCAM and cytokeratins. However, some CTCs do not express these surface antigens, particularly for those of a highly invasive and metastatic property. These invasive tumor cells may lose their epithelial antigens via the epithelial to mesenchymal transition (EMT) process [39].

### 2.2. Biophysical Approach

**Figure 1:**
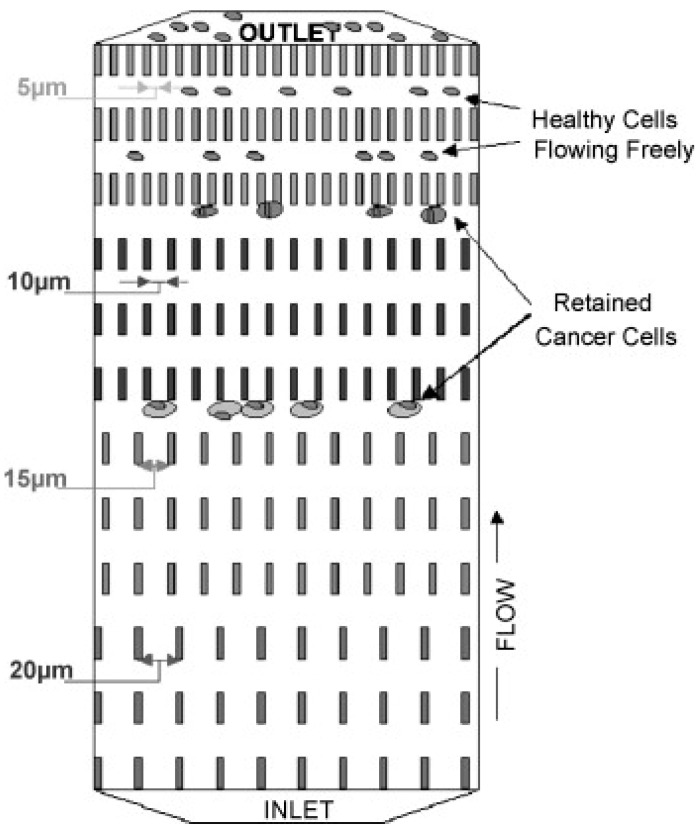
Schematic illustration of the microdevice with successively narrower channels for CTC isolation. Varying channel gap widths (20, 15, 10, and 5 μm) separate cells based on size and deformability. (Reprinted from [41]).

**Figure 2:**
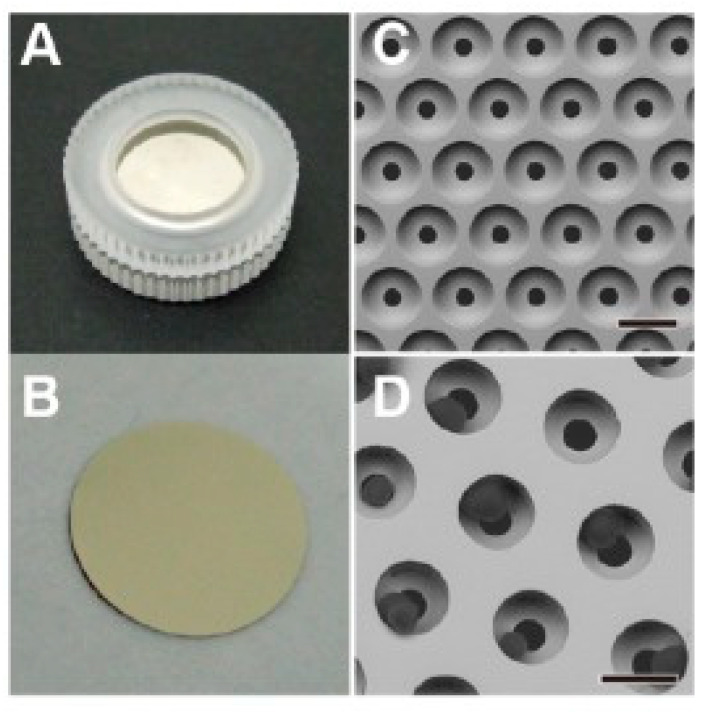
Photographs of the (**A**) 3-dimensional palladium filter cassette and (**B**) filter sandwiched by the upper and lower cassette piece. SEM images of the (**C**) three-dimensional palladium filter and (**D**) tumor cells trapped in the pockets of the filter. (Reprinted from [42]).

Alternatively, the isolation of CTCs can be conducted based on their physical size, deformation, and electrical property [40,41,42,43,44,45,46,47,48]. Because of the mature development of micro-fabrication and microfluidics technology [49,50,51], fabrication of microstructures and control of microfluidic flow were realized for cell-based assays. In the biophysical approach, CTC isolation basically includes the technologies of filtration, hydrodynamics, and dielectrophoresis. For example, successively narrower channels ranging in width from 20 to 5 μm and in depth from 20 to 5 μm were fabricated to retain increasingly smaller cells [41]. A schematic illustration of the microdevice with successively narrower channels is shown in Figure 1. Tumor cells could be isolated from whole blood based on their physical size. Because the cells were isolated by their physical properties, there was no labelling or special treatment on the cells. The intact cells, or DNA could be extracted for molecular analysis. Another example was to use a three-dimensional palladium filter with an 8 μm pore size in the lower layer and a 30 μm pocket in the upper layer to trap CTCs [42], as shown in Figure 2. This microdevice was a simple pumpless device driven by gravity and could enrich CTCs from whole blood within 20 min. The recovery rate of tumor cells from blood was evaluated by cell spike experiments and shown to be over 85%. Living tumor cells could be isolated from the microdevice. In the animal and clinical tests, the number of isolated CTCs from blood significantly increased with the progression of metastasis. The above two examples isolated CTCs using filtration technology. Moreover, some studies adopted hydrodynamics technology to isolate CTCs. Spiral microchannel with inherent centrifugal forces was used for continuous, size-based isolation of CTCs from blood [43]. The recovery rate of over 85% was demonstrated in the experiment of using cancer cell lines. Viable cells could be retrieved by a single step procedure. This approach had realized antibody-independent isolation, high recovery rate (>85%), and high throughput (3 mL/h). On the other hand, based on the polarizability of cells, different cell types experience different dielectrophoretic (DEP) forces. A microfluidic CTC isolation device was developed and cancer cells were isolated by positive DEP force from sample blood cells [26]. The above demonstrations of isolating CTCs were based on their physical properties. However, cells of different cancer types should have different physical properties, e.g., size, stiffness, and density. Thus, the specificity and sensitivity of the CTC isolation from a blood sample should be validated with more clinical studies.

## 3. Microdevices Adopting Single Approach

### 3.1. Single Biochemical Approach

**Figure 3:**
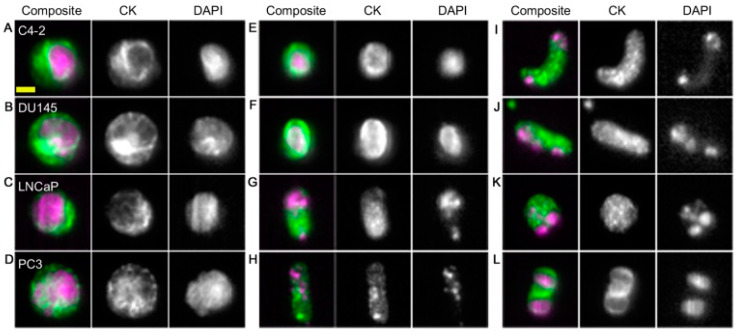
Images of cultured prostate cancer cell (**A–D**) and CTCs from prostate cancer patients (**E–L**) captured using the CellSearch system. (Reprinted from [35]).

**Figure 4:**
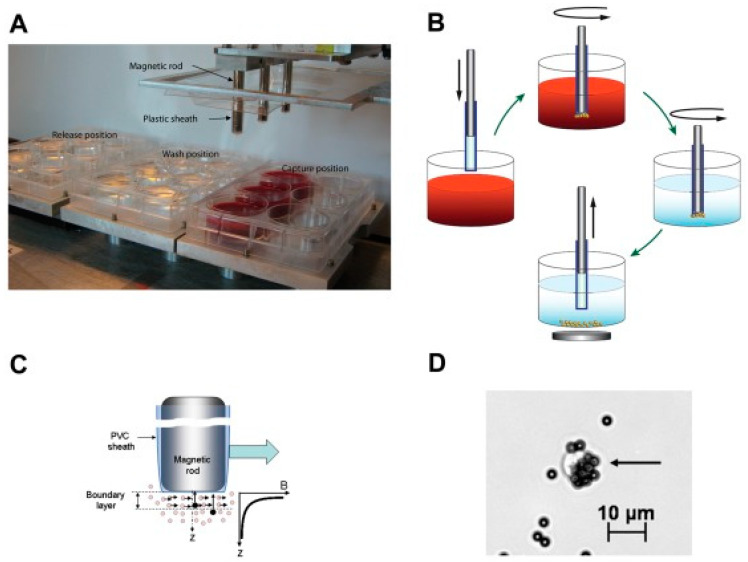
Magnetic sweeper device and cell isolation steps. (**A**) Magnetic sweeper device showing magnetic rods sheathed in plastic above the capture, wash and release stations. (**B**) A diagrammatic view of magnetic sweeper cell isolation protocol. (**C**) A controlled shear force produced by the movement of the magnetic rods in the wash station releases non-specifically bound blood cells. (**D**) Photomicrograph of a CTC labeled with 4.5 μm immunomagnetic beads. (Reprinted from [44]).

**Figure 5:**
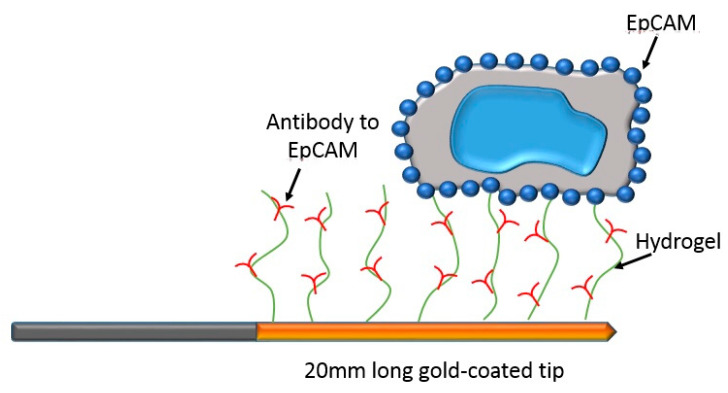
Illustration of the medical wire capturing circulating tumor cells in vivo.

Isolating CTCs based on their surface markers is the most widely used technique currently. The CellSearch system is an FDA-approved platform for prognostic use in various cancers. Magnetic beads conjugated with anti-EpCAM antibodies are used to capture CTCs in blood sample. After collecting the magnetic beads, the CTCs can be isolated by the staining of nuclei and their epithelial structural cytokeratins. Examples of the cancer cells and CTCs staining of nuclei and CK are shown in Figure 3. By using similar magnetic approach, some demonstrations also successfully showed the ability to isolate CTCs [19,20,44,45]. For example, a magnetic sweeper device was developed to improve cell capture efficiency, purity, and throughput to 62%, 51%, and 9 mL/h, respectively [19,44]. The magnetic sweeper device and cell isolation steps are shown in Figure 4. The device was an immunomagnetic cell separator and was a round-bottom neodymium magnetic rod covered with an ultrathin non-adherent plastic sheath. The sheathed rod was robotically driven to sweep through the well containing the targeted cells pre-labeled with magnetic beads. The device was demonstrated to isolate CTCs from all 47 tubes of 9-mL blood samples collected from 17 metastatic cancer patients. Another example was a magnetic sifter device that generates extremely high magnetic field gradients around the edges of magnetic pores [20]. The capture efficiency was as high as 91.4% and the throughput could be at 10 mL/h. On the other hand, a novel approach for in vivo sampling was developed by functionalizing a medical wire with anti-EpCAM antibodies [36,37,38]. An illustration of the medical wire is shown in Figure 5. The medical wire is inserted into the cubital vein for 30 min in order to collect the CTCs. During the insertion, up to 1.5 L of blood is sampled. The medical wire specifically and sensitively catches and enriches CTCs in vivo from circulating peripheral blood. The CTCs are identified and counted by the staining of EpCAM, cytokeratins, and nuclei. In this work, in vivo experiments were conducted to show the suitability, specificity, and sensitivity of this approach. Twenty-four breast cancer or non-small cell lung cancer patients and 29 healthy volunteers were participated. The result showed EpCAM-positive CTCs from 22 of the 24 patients were successfully enriched and no CTCs could be detected in healthy volunteers. The medical wire is a structured medical Seldinger guidewire which is biocompatible and compliant with the regulations for medical devices. It is safe with no noteworthy side effects. This approach is now commercially available, and the device is called GILUPI Cell Collector^®^. Although the recognition of EpCAM for CTC isolation is the mainstream currently, this may miss a subpopulation of metastatic tumor cells that undergo EMT. Thus, the most aggressive cancer cells may actually be the least likely to be captured and identified using this technique.

### 3.2. Single Biophysical Approach

#### 3.2.1. Filtration

**Figure 6:**
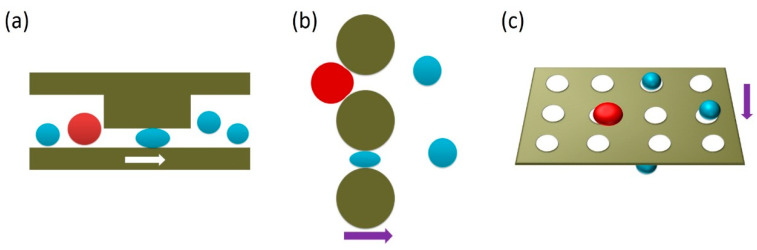
Illustration of the filtering microstructures in microdevices, including (**a**) weir, (**b**) pillar, and (**c**) pore. Red circles represent the CTCs and blue circles indicate the leukocytes, which are relatively small and easily deformed.

**Figure 7:**
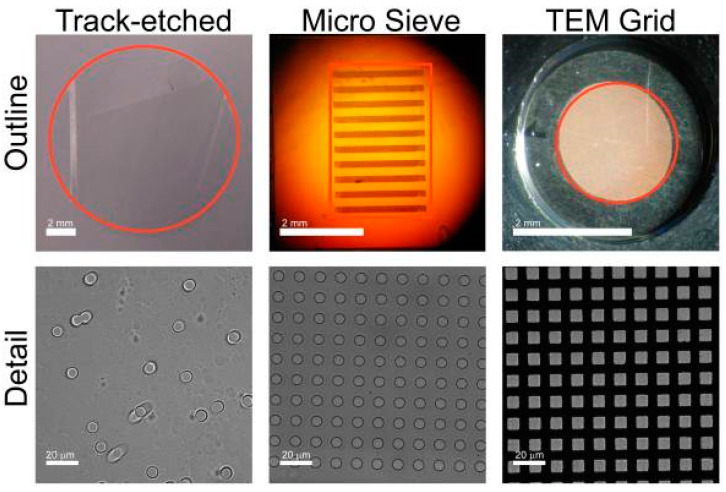
The outline images show a photograph of the track-etch filter, the microsieve and the TEM grid. The perforated area of each filter is indicated in red. The microsieve contains perforated horizontal bars alternated by support bars, giving rise to the horizontal pattern. The detail shows dark field images for the three filters. Spacing of the pores is random for the track-etch filters, leading to occasional double pores. Microsieves and TEM grids have periodical pore spacing. (Reprinted from [56]).

Microdevices with filtering microstructures were developed for isolating CTCs. Filtration is a process of flowing liquid sample through an array of microstructures in order to capture target cells according to their size and deformability. Generally, CTCs which are larger and stiffer are retained while most blood constituents are removed. The filtering microstructures are generally categorized into three types, including weir [52], pillar [53], and pore [22], as shown in Figure 6. The microstructure of weir microstructures could be a microchannel formed between a textured surface and a flexible membrane [52]. The textured surface consisted of an array of micro-scale pockets. As cells flowed through the microchannel, the velocity of the larger cells was attenuated relative to the smaller cells. The smaller cells could freely pass through the microchannel, while the larger cells were trapped by the micro-scale pockets. The microstructure of pillar could be placed in a microchannel and captured the CTCs which are generally larger and stiffer [53]. The pillar microstructures should be optimized by computational analysis to enhance the isolation efficiency. The pore microstructures could also enrich viable CTCs from the blood [22]. A microdeive was developed and consisted of top and bottom parylene membranes with pores. The locations of the pores were shifted between two membranes. The bottom membrane supported the captured cells in order to minimize the stress concentration on cell membrane. Since CTCs are generally larger and stiffer compared to other circulating cells in blood samples, the microdevices with filtering microstructures can accomplish label-fee CTC isolation. After filtration, the CTCs were generally identified under microscope by fluorescent staining [54]. It reported that the nuclear to cytoplasmic ratio and cell size are highly different between the CTCs and blood cells. On the other hand, a clinical study reported the correlation between the number of CTC and disease stage and progression [55]. In this study, the CTCs were enriched from the nucleated cell fraction by filtration. The cell suspension was transferred into a 20 mL syringe and filtered through an 8 μm polycarbonate filter attached to the syringe. Then, the CTCs were enumerated visually following CK8 immunostaining. In 131 breast cancer patients, there was a higher incidence of CTC in patients with distant metastatic than those with node-positive, or node- negative disease. Moreover, another study evaluate different filter types for CTC enrichment [56]. In this study, whole blood spiked with cells from 9 tumor cell lines were respectively passed through polycarbonate track-etched filters with a pore diameter of 5, 8, and 10 μm (Whatman Nucleopore), silica nitride microsieves with a pore diameter of 5, 6, 7, 8, 9, and 10 μm (Aquamarijn), and copper transmission electron microscopy (TEM) grids with 7.5 μm square pores (Gilder Grids), as shown in Figure 7. The result reported that the 8 µm track-etched filter and the 5 µm microsieve had the best performance on MDA-231, PC3-9 and SKBR-3 cells, enriching >80% of cells from whole blood. The TEM grids had poor recovery of ∼25%. In addition, Gascoyne et al. measured the diameter of CTCs from patients with castrate-resistant prostate cancer. They found that the average size was ranging from 7.05 to 8.94 μm [35]. In comparison, the leukocyte has a typical diameter ranging from 6 to 9 μm [57]. The cell sizes between CTCs and leukocytes are highly overlapped. That indicates tumor cell types have significant difference in physical properties, e.g., size and stiffness. Thus, the specificity and sensitivity of the CTC isolation based on filtration may require to be validated with more clinical studies.

#### 3.2.2. Hydrodynamics

**Figure 8:**
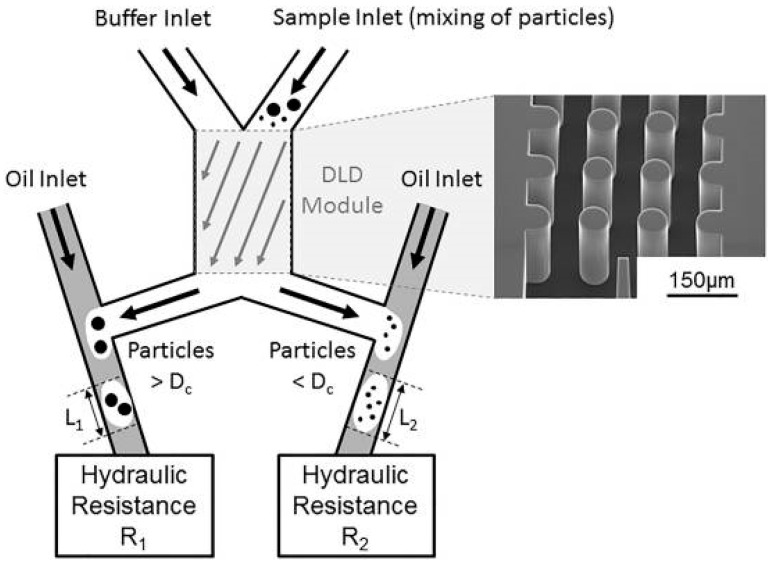
Particles larger than the critical diameter (D_c_) were deviated towards the left outlet, while smaller particles flowed in the right outlet. The DLD outlet was connected to a T-junction with an oil inlet, for generation of droplets of lengths L_1_ and L_2_. (Reprinted from [58]).

**Figure 9:**
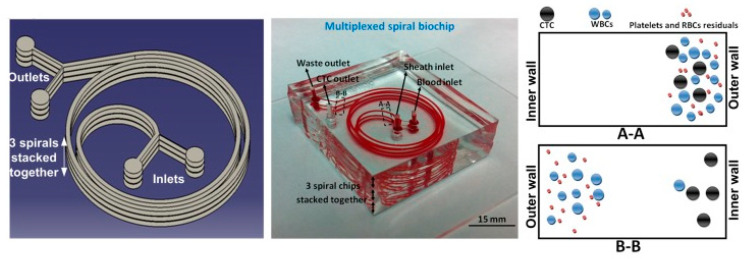
Illustration and photograph of the multiplexed spiral microdevice. (Reprinted from [63]).

As the mature development of microfluidic technology, precise control of flow stream using complicated microstructures could be achieved to isolate biological cells. Hydrodynamic separation of the cells was demonstrated on the interaction between particles and obstacles [23,58] and inertial effect [25,59,60,61,62,63,64]. For example, crescent-shaped trap arrays with a fixed gap of 5 μm in width were developed to enrich CTCs from whole blood [23]. It is a label-free approach to isolate CTCs based on their distinctively different deformability and size. A capture efficiency of >80% was reported on breast and colon cancer cells. Another example was to use deterministic lateral displacement (DLD) to separate two populations of particles around a specific size [58]. DLD is a size-based fractionation technique. Fluid path of particles in different sizes is oriented by an array of regularly displeased pillars. A platform shown in Figure 8 was developed and consisted of T-junctions connected to the DLD outlets. Particles in different sizes were sorted and encapsulated in droplets. Moreover, a number of studies adopted fluid inertia for biological applications [59,60,61,62,63,64]. Inertial microfluidics works in the intermediate Reynolds number region and was demonstrated on precise fluid manipulation. Due to its high throughput, simplicity, and inexpensiveness, inertial microfluidics is a promising tool for cellular processing. A multiplexed spiral microdevice was developed to enrich CTCs from blood samples [63]. The microdevice is shown in Figure 9. A blood sample and sheath fluid were introduced to the inlets of the microdevice by two syringe pumps. Because of the inertial lift and drag forces, CTCs moved near the inner wall of the microchannel and blood cells went towards the outer wall of the microchannel. Thus, the CTCs could be separated from the blood sample. Moreover, a multistage microfluidic device was developed to separate cancer cells from red blood cell suspension using inertial migration forces [64]. The collection efficiency and enrichment of cancer cells could reach 85% and 120-fold. This microdevice was able to effectively remove red blood cells up to 1% hematocrit condition with a throughput of 565 μL/min. Since hydrodynamic separation is a non-label and high-throughput approach, it is possible for the development of CTC isolation device.

#### 3.2.3. Dielectrophoresis

**Figure 10:**
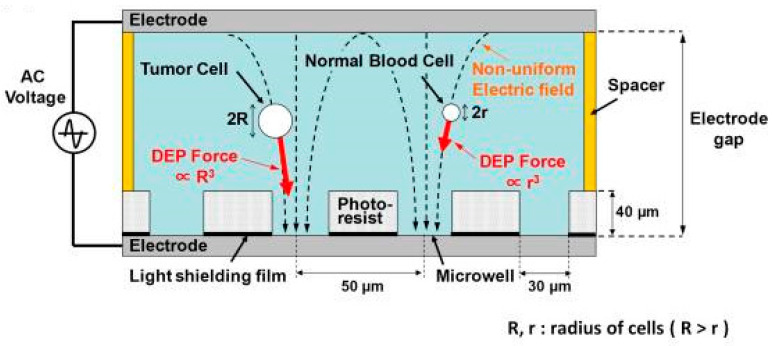
Schematic image of capturing cells using DEP force. (Reprinted from [66]).

**Figure 11:**
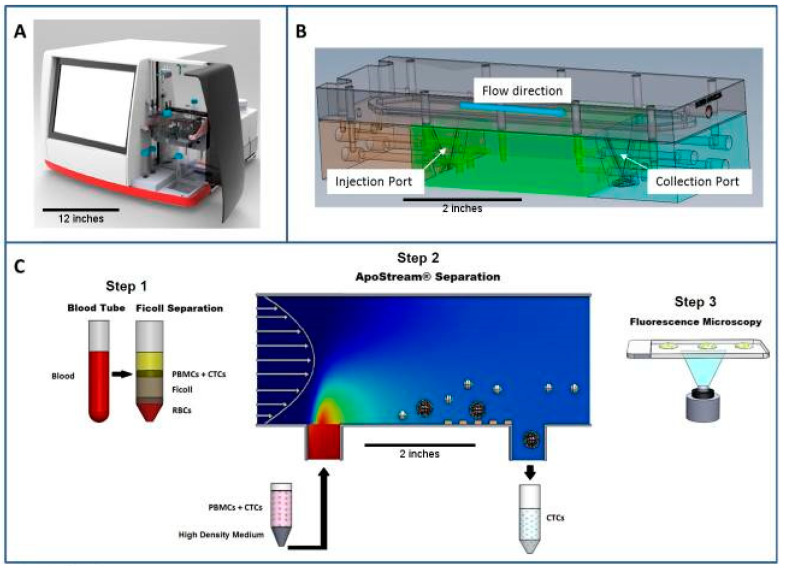
Photography and illustration of the ApoStream^®^ instrument. (**A**) The ApoStream^®^ prototype instrument. (**B**) Illustration of the flow chamber showing V-shaped injection and collection ports. (**C**) Step 1: Sample processing by Ficoll density gradient separation to isolate blood cells and CTCs. Step 2: Dielectrophoresis (DEP) enrichment starting with sample injection, ion diffusion, DEP separation of CTCs from blood cells, and CTC collection. Step 3: Downstream analysis using immunofluorescence or other techniques for CTC identification and enumeration. (Reprint from [69]).

Dielectrophoresis (DEP) is a phenomenon by which a polarized particle is forced to move under a non-uniform electric field [65]. Based on the DEP phenomenon, biological cells can be manipulated selectively. The magnitude and direction of DEP force acting on a cell depend on several factors, such as cell membrane, cytoplasm-charge, and cell size. The electric field induces charges inside a cell to form dipoles. When the cell is more polarizable than the suspending medium, it is attracted towards the region of high electric field. This motion is called positive DEP. Conversely, negative DEP is defined as when the cell is less polarizable than the suspending medium, and it is repelled from the region of high electric field [66]. A schematic image of capturing cells using DEP force is shown in Figure 10. A non-uniform electric field can be generated between the upper and lower electrodes. The DEP force exerts on a cell and the cell is manipulated towards the microwell. On the other hand, interdigitated microelectrode was used to generate non-uniform electric field for inducing DEP force. An interdigitated microelectrode array was developed to dielectrophoretically isolate breast cancer cells from spiked healthy donor blood [67]. The dielectric properties of metastatic human breast cancer cell line MDA231 are significantly different from other blood cells. The positive DEP force generated by the microelectrode array attracted the tumor cells and other cells flowing through the microdevice. Upon removal of the DEP force, the tumor cells were collected with a capture efficiency of 95%. Based on the similar approach, Huang et al. developed a microdevice with a thin chamber in which the bottom wall embedded an array of microelectrodes [68]. DEP force generated by the microelectrodes levitated cells suspended in the chamber and affected their equilibrium heights. Based on the balance of dielectrophoretic, gravitational, and hydrodynamic lift forces, tumor cells were demonstrated to separate from peripheral blood mononuclear cells. Another example of cell isolation by DEP phenomenon is the ApoStream™ instrument [26,69]. Photography and an illustration of the ApoStream^®^ instrument are shown in Figure 11. The instrument was composed of a flow chamber with electrodes fabricated on its floor. The DEP field was generated by the electrodes in the flow chamber. Cancer cells were introduced to the chamber at the upstream end. When the cells encountered the DEP field, the DEP forces pulled cancer cells towards the chamber floor and repelled other cells. This work demonstrated the ability to achieve a recovery rate of around 70% from cancer cells spiked into whole blood. The collected cells were still viable, with a viability of 97.1%. The DEP approach can isolate viable cells independent of their EpCAM expression level. It has the potential to apply to a wide range of cancer types.

## 4. Microdevices Adopting Multiple Approach

### 4.1. Multiple Biochemical Approach

The CTC isolation microdevices based on the recognition of single CTC-specific marker, e.g., EpCAM, were discussed. However, the binding of the EpCAM is naturally slow and it becomes a hurdle for high-throughput clinical applications. A biomimetic surface functionalized with selectin and anti-EpCAM was created to study the affinity responses of leukocytes and CTCs [70]. Leukocytes and CTCs exhibited rolling on selectin-immobilized surfaces under fluid flow. The rolling velocity of CTCs was faster than that of leukocytes. However, the CTCs could bind to the anti-EpCAM-coated surface under flow. By the combination of the rolling and binding property, it resulted in substantially enhanced CTC capture efficiency. Thus, the microdevice could adopt this affinity property to enhance the specificity and sensitivity of CTC isolation. A microdevice consisting of a 300 μm microtube coated with selectin and CTC-specific antibodies was developed [27]. Selectin is a highly specific marker for leukocytes. When leukocytes flowing in the blood stream roll on the endothelium, under certain stimulation, they will firmly bind to the endothelial selectin and subsequently extravasate. Because the CTCs exhibit the same process to metastasize to tissue, the microdevice can isolate the CTCs via the biomolecular surface at high flow rates. The ability to isolate 20-704 CTCs per 3.75 mL of clinical blood sample was demonstrated at a flow of 4.8 mL/h. The device achieved a capture efficiency of ~50% and an average purity of 66%.

### 4.2. Multiple Biophysical Approach

**Figure 12:**
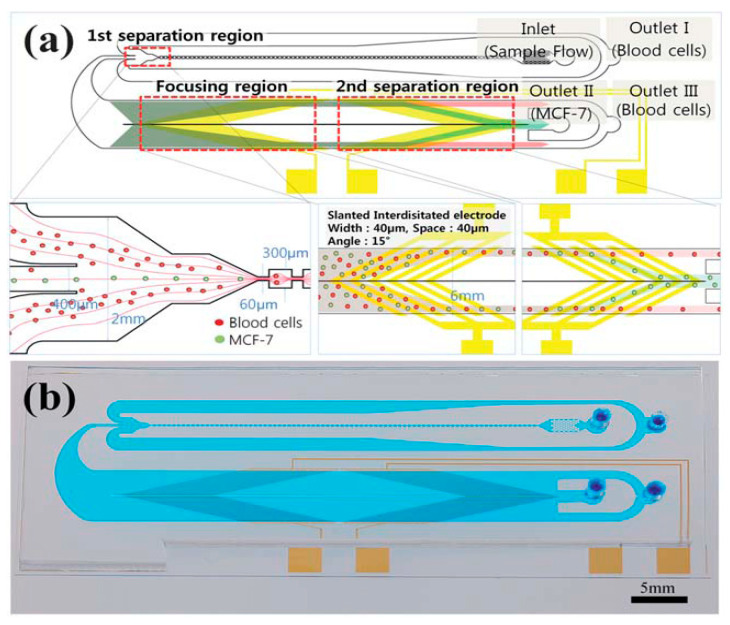
(**a**) Schematic diagram of the microdevice for cell separation using multi-orifice flow fractionation (MOFF) and DEP. In the first separation region, the relatively larger cancer cells and a few blood cells passed through the center channel and entered the DEP channel, after which most blood cells exited through outlet I. In the focusing region, all cells experienced a positive DEP force and then aligned along both sides of the channel. Finally, the second separation region selectively isolated cancer cells via DEP. (**b**) Photography of the fabricated microdevice. (Reprinted from [28]).

In order to enhance the CTC isolation efficiency, the techniques of microfluidic flow control and dielectrophoresis were adopted. The microdevice consisted of serially integrated multi-orifice flow fractionation (MOFF) and the dielectrophoretic mechanism [28]. The MOFF element was composed of an inlet, a filter, a multi-orifice segment, a fraction segment, and two wide outlets. The multi-orifice segment consisted of an alternating series of contraction channels and expansion chambers. The total length of the multi-orifice segment was about 36 mm and contains 80 repeated expansion elements. The central channel was for the larger cells (CTCs), with a 400 mm channel width and a link to outlets II and III via DEP separation. The other two side channels, with the width of 760 mm, collected the smaller cells (blood cells) and released them through outlet I, as shown in Figure 12. The microdevice combining these two different techniques enabled a high-speed continuous flow-through isolation without labeling. The results reveal a 162-fold increase in cancer cells at a 126 μL/min flow rate. The blood cells were efficiently removed with a separation efficiency of >90%.

### 4.3. Combined Biochemical and Biophysical Approach

A microdevice was embedded with 78,000 silicon micropillars functionalized with antibodies targeting EpCAM to allow direct processing of whole blood [29]. The geometry of the micropillars provided an abundant total surface area (970 mm^2^) for the interaction between micropillars and cells. A capture efficiency of ≥60% and a final sample purity of about 50% were achieved when processing at a throughput of 2.5 mL/h. In order to further enhance the capture surface and collision opportunity, microvortices generated by herringbone microstructures were induced in the second generation of the microdevice [71]. The EpCAM-specific capture antibodies were immobilized to the herringbone-shaped grooves along the bottom surface of the microdevice, resulting to the increase in cell and surface contact based on the laminar flow pattern. This could improve the capture efficiency to 91.8%. Moreover, micropillar approach was also demonstrated on the capture of prostate CTCs [30]. Micropillars were coated with antibodies targeting prostate-specific membrane antigen (PSMA). This microdevice achieved a capture efficiency of 85% and purity of 68%, and identified CTCs from 18 of 20 prostate cancer patient samples. Furthermore, nanostructured substrates were employed in microdevice to have an extremely high contact surface area for immunoaffinity [72]. Silicon nanopillars conjugated to anti-EPCAM and chaotic micromixing were integrated to achieve a capture efficiency of >95% from blood at an optimal throughput of 1 mL/h.

**Figure 13:**
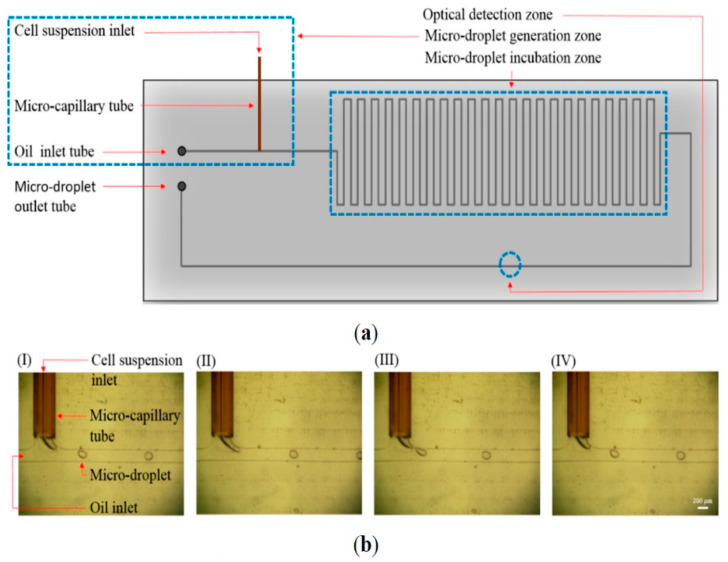
(**a**) Schematic illustration of the microdevice (top-view layout). (**b**) Photographs of continuous micro-droplet generation process ((I)-(IV)) (Reprinted from [74]).

Alternatively, microdevices embedded with biosensors were developed for identifying an extreme rare number of CTCs in blood samples. For example, a high throughput optical sensor with fiber-optic array scanning technology was capable of analyzing 300,000 cells per second [73]. It is promising for CTC detection as they are less vulnerable to cell loss. In addition, microfabricated Hall effect sensors were developed to detect CTCs with an assay throughput of ~10^7^ cells/min [74]. The sensors employed a hybrid microfluidic/semiconductor microdevice to maximize cellular detection across a fluidic stream. Eight sensors were arranged into an overlapping 2 × 4 array. CTCs were captured by magnetic nanoparticles conjugated to antibodies targeting various antigens, i.e., EpCAM, HER2, EGFR, and MUC1. Then, they flowed through an array of sensors that measure Hall voltages induced by the magnetic flux of each labeled cell. Furthermore, a microfluidic-based optical sensing device for label-free detection of CTCs through their lactic acid production [75]. The microdevice consisted of a micro-droplet generator, a micro-droplet incubator, and an optical detection zone. An illustration of the microdevice and photographs of continuous micro-droplet generation process are shown in Figure 13. The cells encapsulated in micro-droplets supplemented with fluorescence-based lactate reagent were continuously delivered to the microchannel (OD: 375 μm and ID: 50 μm). Since CTCs generate more lactate acid then normal cells, quantification of lactic acid through the fluorescence-based optical sensing could immediately identify the CTCs in the micro-droplet. The detection signal was proportional to the number of CTCs and insensitive to the leukocytes within the micro-droplet. This novel approach opens a new route to detect live CTCs without labeling.

## 5. Discussion and Conclusions

A commercial system called the CellSearch^®^ system using the technology of magnetic beads was launched for the detection and enumeration of CTCs. The CTCs suspended in three-dimensional space are captured by magnetic beads conjugated with CTC-specific markers. This highly improves the detection limit, especially for targets in extremely low concentrations. The enumeration of CellSearch^®^-enriched CTCs has been established as a prognostic marker and a predictor of patient outcome in metastatic cancers. Currently, the CellSearch^®^ system is the only FDA-approved equipment for clinical CTC diagnostics in which a positive enumeration is associated with decreased progression-free survival and overall survival for cancer patients. It is the gold standard for CTC enumeration and the only one that has been both analytically and clinically validated. However, it still has the limitation of sensitivity and is not applicable to all types of cancer.

Almost all existing biochemical isolation processes discriminate CTCs from hematological cells using antigens expressed in epithelial cells. Since normal epithelial cells rarely circulate in peripheral blood, cells isolated using these markers are assumed to be CTCs. A fundamental flaw with the existing biochemical approach is that a subpopulation of metastatic tumor cells are likely to undergo EMT, which is associated with a loss of expression for epithelial markers, such as EpCAM and CK. Consequently, the most aggressive cancer cells may actually be the least likely to be captured and identified using this technique.

Biophysical isolation methods rely on differences in the physical properties of CTCs compared to leukocytes, including cell size, shape, deformability, density, electrical polarizability, and magnetic susceptibility. These methods are label-free and prevent the prejudice of epithelial antigen in the existing biochemical methods. Microfiltration enables extremely high throughput processing of full tubes of blood within minutes. However, the overlap in size distribution between CTCs and large leukocytes results in sample purities of less than 10%. It is also possible that smaller CTCs or CTC fragments may be missed. Compared with filtration and hydrodynamic methods, DEP-based methods currently lag in performance in both selectivity (enrichment over leukocytes typically < 100) and throughput (typically <1 mL/h). In addition, the DEP-based technology has not yet thoroughly evaluated with clinical samples, so its clinical utility must be determined.

The isolation of CTCs has been a hot research topic for decades. In order to develop effective CTC enumeration and isolation methods, various microdevices have been developed to capture CTCs. Most of the microdevices used silicon or polymer materials such as PDMS and PMMA as the device substrate. These materials are low cost and have excellent optical transparency. However, they are unable to overcome the bottleneck to enhance the capture efficiency, enrichment, purity, and throughput. In the past decade, a new discipline of using nanomaterials was arisen in order to find a new direction of CTC isolation technology [76]. Magnetic nanoparticles have the advantages of cellular internalization, signature size-based, and easy scale-up [77,78,79]. Vertically aligned carbon nanotubes have the advantages of increasing internal and external surface area, cell-nanotube and fluid-nanotube interactions, and conductivity [80,81]. Nanopillars, nanowires, or nanofibers have the advantages of promoting interactions with extracellular features, increasing surface area, and enhancing the thermosensitivity [72,82]. Nanoroughened surfaces have the advantages of promoting interactions with extracellular features, increasing surface area, and enhancing antigen-independent capture [83,84]. Graphene oxide has the advantages of promoting interactions with extracellular features and increasing surface area and conductivity [85,86]. In the future, we expect that the efficiency of CTC isolation can be improved by the use of nanomaterials to capture CTCs based on their physical properties.

CTCs are recognized as liquid biopsies and closely correlate with progression free and overall survival of cancer patients. Enumeration of CTCs in bloodstream is a very important indicator to estimate the therapeutic outcome in various metastatic cancers. Thus, CTC isolation is one of the most popular research areas in biomedical microdevices. The commercial success of CTC isolation requires a robust technology with cost-effective and high-throughput capabilities. Moreover, by acquiring the CTCs from patients, genomic mutation can be analyzed to identify potential targeted therapies. Therefore, CTC isolation and analytical technologies will eventually be integrated into clinical patient care to assist cancer treatment decision. The isolation technologies, analytical assays, and cancer management protocols are required to be standardized and reproducible in order to establish a total therapeutic strategy for cancer patients.

## Figures and Tables

**Table 1:** Summary of circulating tumor cells (CTC) isolation microdevices.

Category	Isolation Principle
Microdevices adopting single approach	
Biochemical approach	- CTC-specific marker
Biophysical approach	- Filtration - Hydrodynamics - Dielectrophoresis
Microdevices adopting multiple approach	
Multiple biochemical approach	- Selectin and CTC-specific marker
Multiple biophysical approach	- Microfluidic flow control and dielectrophoresis
Combined biochemical and biophysical approach	- Microstructures functionalized by CTC-specific markers - Biosensors

**Table 2:** Summary of the efficiency, purity, and throughput of CTC isolation microdevices.

Isolation Principle	Capture Efficiency	Isolation Purity	Throughput	Ref.
CTC-specific marker	62 ± 7%	51 ± 18%	9 mL/h	[19]
	91.4%	17.7 ± 9.3%	10 mL/h	[20]
Filtration	89 ± 9.5%	-	<10 min	[21]
	86.5 ± 5.3%	-	12–20 mL/h	[22]
Hydrodynamics	>80%	-	0.7 mL/h	[23]
	~85%	-	-	[24]
	~80%	-	1.2 mL/h	[25]
Dielectrophoresis	~70%	-	7.5 mL/h	[26]
Selectin and CTC-specific marker	~50%	66 ± 3.9%	4.8 mL/h	[27]
Microfluidic flow control and dielectrophoresis	>90%	162-fold	126 μL/min	[28]
Microstructures functionalized by CTC-specific markers	≥60%	~50%	2.5 mL/h	[29]
	85 ± 5%	68 ± 6%	1 mL/h	[30]
